# 51152 Efficacy of Bimodal Visual-Olfactory Training in Patients with COVID-19 resultant Hyposmia or Anosmia Using Patient-Preferred Scents (VOLT Trial - Visual-OLfactory Training)

**DOI:** 10.1017/cts.2021.711

**Published:** 2021-03-30

**Authors:** Amish Mustafa Khan, Dorina Kallogjeri, Jay F. Piccirillo

**Affiliations:** Washington University School of Medicine

## Abstract

ABSTRACT IMPACT: Olfactory dysfunction is a defining symptom of COVID-19 infection. As the number of total, confirmed COVID-19 cases approaches 7 million in the United States, it is estimated that there will be up to 500,000 new cases of chronically diminished smell. We offer a promising treatment. OBJECTIVES/GOALS: The primary aim is to explore the main effects and interaction of bimodal visual-olfactory training and patient-preferred scents on olfactory training in patients with post-COVID-19 hyposmia or anosmia. METHODS/STUDY POPULATION: The study will utilize a 2x2 factorial design. The two effects we will explore are unimodal versus bimodal training and conventional versus patient-preferred odors. All 4 arms will undergo 12 weeks of olfactory training. Participants will be assessed pre and post-intervention. Measurements of olfactory function include the objective smell identification test and subjective measures including the Clinical Global Impression Scale and Olfactory Dysfunction Outcomes Rating. Individuals eligible for the study include men and women between 18 and 70 years of age with olfactory dysfunction of at least 3 months duration initially diagnosed within 2 weeks of a COVID-19 infection. Of note, we will enroll nationally. RESULTS/ANTICIPATED RESULTS: We anticipate that the bimodal, patient-preferred scents training group will have the greatest improvement in smell scores, number of responders, and patient-reported sense of smell and health-related quality of life due to an additive interaction between the bimodal visual-olfactory and patient-preferred interventions. DISCUSSION/SIGNIFICANCE OF FINDINGS: The pathophysiology of COVID-19 olfactory dysfunction is mediated through damage to the peripheral and central olfactory pathways. This suggests that interventions most likely to be efficacious target both pathways, as olfactory training does.

